# Number Agreement Attraction in Czech Comprehension: Negligible Facilitation Effects

**DOI:** 10.1162/opmi_a_00107

**Published:** 2023-10-27

**Authors:** Jan Chromý, Radim Lacina, Jakub Dotlačil

**Affiliations:** Institute of Czech Language and Theory of Communication, Faculty of Arts, Charles University, Prague, Czech Republic; Institute of Cognitive Science, Osnabrück University, Osnabrück, Germany; Department of Linguistics, University of Potsdam, Potsdam, Germany; Department of Languages, Literature and Communication, Utrecht University, Utrecht, The Netherlands

**Keywords:** agreement, attraction, sentence processing, interference, syncretism, Czech

## Abstract

Number agreement attraction in comprehension has been extensively studied in various languages and it has been claimed that attraction effects are generally present across languages. In this paper, four experiments on Czech are presented, each examining a different structure. The Bayesian hierarchical models and Bayes factor analysis pointed towards no agreement attraction effects in three of the experiments. Only in one experiment an effect interpretable as signaling agreement attraction was observed. Its size, however, was so small that it did not translate into a clear preference for models with agreement attraction. The data from the four experiments were further compared to available data from several other languages (English, Armenian, Arabic, and Spanish). The emerging picture is that in Czech, agreement attraction effects are negligible in size if they appear at all. This presents a serious challenge to current theoretical explanations of agreement attraction effects.

## INTRODUCTION

When exposed to speech or written text, our minds swiftly process the incoming information within a span of several hundred milliseconds. This cognitive ability is truly remarkable, considering the multitude of tasks that comprehenders must undertake, including word recognition and syntactic parsing (Futrell et al., 2020; Weber & Scharenborg, 2012). An important inquiry arises regarding the extent of variation in these tasks among different languages or, more precisely, among speakers of diverse languages. Examining the comprehension processes of typologically distinct languages thus provides a valuable opportunity for cognitive science, as it allows us to investigate how general cognitive mechanisms (such as memory) interact with language-specific constraints. In this particular study, we focus our attention on a specific phenomenon–agreement attraction–in the Czech language. We demonstrate that, in Czech, this effect is either negligible or completely absent which is surprising, since it has been attested in various other languages. Furthermore, we discuss the implications of this finding in relation to theories that rely on domain-general memory mechanisms.

In language processing, speakers as well as comprehenders must often deal with establishing long-distance dependencies between non-adjacent elements of a sentence. Chief among these phenomena is agreement (Corbett, 2003). It has long been known that the processing of agreement can sometimes not be fully successful. In production, speakers sometimes make speech errors such as the following (Bock & Miller, 1991):

(1)  *The cost of the improvements *have* not yet been estimated.

In sentence (1), the auxiliary ought to agree with the subject head *the cost*. However, its form *have* betrays the establishment of an erroneous agreement relation with the plural-marked NP *the improvements* in the course of production. This phenomenon has been termed agreement attraction or proximity agreement (the latter term has been used in traditional linguistic discourse, see Francis, 1986, or Quirk et al., 1985).

When it comes to comprehending sentences such as (1), it has been claimed that agreement attraction effects are also present. For example, researchers have found that when comprehenders are asked to give binary ratings of the acceptability of these sentences under time pressure, they often come under the illusion that they are in fact grammatical (Hammerly et al., 2019; Wagers et al., 2009). But crucially, agreement attraction effects have also been documented in online processing (using the self-paced reading paradigm). Consider sentences such as (2)–(5) (taken from Pearlmutter et al., 1999):

(2)  The key to the cabinet was rusty from many years of disuse.
(3)  The key to the cabinets was rusty from many years of disuse.
(4)  *The key to the cabinet were rusty from many years of disuse.
(5)  *The key to the cabinets were rusty from many years of disuse.

The sentences (2) and (3) are both grammatical and they only differ in the number feature of the NP that stands between the subject (*the key*) and the verb (*was*). We will call the non-subject NP (i.e., *the cabinet*/*the cabinets* in the example above) *the attractor*. The sentences (4) and (5) are ungrammatical, since the subject and the verb do not agree in number and thus, general agreement rules are violated. These two sentences also differ only in the number of the attractor noun (*cabinet* or *cabinets*). Agreement attraction effects are manifested in the facilitatory interference of the plural attractor on the plural verb in such ungrammatical sentences. The facilitatory interference is, among other things, observable in comprehension studies, which report faster reading times of the verbal and/or postverbal regions in ungrammatical sentences with the plural attractor like (5), compared to the reading times of the same regions in ungrammatical sentences with the singular attractor, (4).

In the following sections, we first critically summarize the evidence for agreement attraction effects in the comprehension of various languages. Then, we will discuss the most prominent theoretical explanations of the agreement attraction effects. Lastly, we will discuss the aims of the current study, in which we test agreement attraction effects in the comprehension of Czech.

### Generality of Agreement Attraction Effects

Agreement attraction is arguably one of the cross-linguistically most widely studied phenomena in the domain of L1 language processing research. Besides English (e.g., Cunnings & Sturt, 2018; Laurinavichyute & von der Malsburg, 2022; Parker & An, 2018; Tanner et al., 2014; Wagers et al., 2009), agreement attraction effects in comprehension have been attested in French (Franck et al., 2015; Franck & Wagers, 2020), Spanish (Acuña-Fariña et al., 2014; Lago et al., 2015), German (Lago & Felser, 2018), Arabic (Tucker et al., 2015, 2021), Russian (Slioussar, 2018), Armenian (Avetisyan et al., 2020), Turkish (Lago et al., 2019; Turk & Logačev, 2021) and Greek (Paspali & Marinis, 2020). The existence of agreement attraction effects (i.e., the interference of plural attractor nouns resulting in the facilitation of processing ungrammatical plural verbs) has been also supported by a meta-analysis (Jäger et al., 2017).

In sum, the evidence for agreement attraction effects is solid and has even lead to claims that agreement attraction is a universal phenomenon due to the generality of the underlying memory mechanism that has been argued to be the cause of the effect (cf. Lago et al., 2015).

However, there are several reasons to believe that the idea of the cross-linguistic generality of the agreement attraction effects in comprehension might not be tenable.

First, the degree to which agreement attraction can be observed in natural contexts differs across languages. In English, the phenomenon is relatively frequent (Francis, 1986) which is also reflected in the fact that it is described in detail by English grammar books (Quirk et al., 1985, pp. 757–759). In contrast, in Czech, number agreement attraction seems to be almost completely absent in ordinary discourse. To demonstrate this briefly, we examined the corpus SYN2020, a representative and reference corpus of contemporary written Czech, which is morphologically tagged and contains about 100 million text words (Křen et al., 2020). We examined all occurrences of nouns in the nominative singular (i.e., the subject) followed by a preposition, a plural noun (i.e., the attractor) and a verb with all these words being part of the same sentence^1^. In other words, we looked for structures similar to (3) or (5) (the only difference being that Czech does not have articles, so the query did not contain them). We found 7088 occurrences out of which 6820 contained a singular verb (i.e., the agreement was correct) and 268 contained a plural verb (i.e., possible examples of agreement attraction). Having manually checked the latter group, we concluded that only three cases could be considered genuine occurrences of agreement attraction (the rest were typically sentences with coordinated subjects in which the plural verb was grammatical, or even more complicated structures). These data are based on a written corpus of Czech and it may be so that in spoken Czech, the situation is different. We thus examined the largest corpus of Spoken Czech (ORAL v1; Kopřivová et al., 2017) using a similar query to the one for the SYN2020 corpus. We found only 357 occurrences of nouns in the nominative singular followed by a preposition, a plural noun and a verb. Only in five cases, the plural verb was used and none of these was an example of agreement attraction. Admittedly, this corpus is relatively small for such an analysis (it contains 5,368,392 tokens, not counting punctuation marks or comments), but it seems to reflect the very same trend as in the written corpus, namely, that number agreement attraction seems to be an extremely rare phenomenon in Czech. This stands in clear contrast to English (Francis, 1986). Such large differences in the frequency of agreement attraction in production cast doubts on the cross-linguistic generality of agreement attraction.

Second, to our knowledge, carefully controlled cross-linguistic studies on agreement attraction in comprehension have so far not been conducted (with the exception of Chromý et al., 2023 which was done in parallel to the present study and documented a clear difference between Czech and English using a direct experimental comparison). The claims about the cross-linguistic status of agreement attraction are thus generally based on separate studies on different languages which differ in many aspects other than only the language being tested (such as the number of experimental stimuli, their lexical content and their syntactic structure, number and the structure of filler items, form and frequency of comprehension questions, instructions given to participants etc.). The evidence in favor of the cross-linguistic presence of agreement attraction is indeed solid considering the previously published studies. However, an unresolved and intriguing question is whether the magnitude of the agreement attraction effects is similar in different languages or whether the size of the effect systematically varies between languages. It may well be that in some languages, agreement attraction effects are relatively pronounced whereas in others, such effects are rather weak or even negligible.

Third, while considering the claims of cross-linguistic generality of agreement attraction, methodological issues are especially important. As already mentioned, there is a problem of the comparability of the effects found in studies on different languages. However, other issues are crucial for the credibility of the results for each language. In an elaborate meta-analysis, Jäger et al. (2017) argue that to obtain reasonable statistical power to assess interference effects of the magnitude of 28 ms with standard deviations of 100 to 300 ms, testing around 100 or 120 participants in an experiment ought to be the minimum. Unfortunately, many of the studies cited at the beginning of this section are far from having such sample sizes. From this perspective, many studies on agreement attraction could be considered underpowered which means that there is a relatively high risk of Type S and Type M errors (Gelman & Carlin, 2014) in the literature. While still informative, these studies cannot be relied upon to deliver strong evidence in favor or against substantial theoretical claims concerning the cross-linguistic status of agreement attraction effects. As we already acknowledged, agreement attraction is a cross-linguistically relatively well-studied phenomenon—at least in comparison to other issues in psycholinguistics. However, to claim that agreement attraction effects are cross-linguistically uniform (or even universal), one would need substantially more evidence than currently at hand.

Fourth, even for a single language, number agreement attraction effects have been attested only in certain environments. For example, Parker and An (2018) (following Van Dyke, 2011) showed a difference in interference effects between attractors that played different grammatical functions.

Parker and An (2018) argue that interference effects are mediated by the argument status of the attractor and that the reason for this is that the core arguments are encoded more distinctly in memory than oblique arguments (cf. Chromý & Vojvodić, 2023).

It also seems that the typological structure of a language may impose certain constraints on agreement attraction. Slioussar (2018) examined agreement attraction in Russian, which is a highly inflectional language with overt case marking, and found facilitatory interference only in those cases where the form of the attractor was homonymous (i.e., syncretic) with the nominative plural as in example (6), even though it actually carried the singular feature. If the form of the attractor was not homonymous with the nominative plural form, such as in example (7), no agreement attraction effects were documented.

(6)  Komnat-a  dlja večerink-i     byl-i prostorn-ymi   …
    room-nom.sg for party-gen.sg=nom.pl were spacious-instr.pl  …
    ‘The room for the party were spatious …’
(7)  Komnat-a  dlja večerinok     byl-i prostorn-ymi   …
    room-nom.sg for party[gen.pl≠nom.pl]  were spacious-instr.pl  …
    ‘The room for the parties were spatious …’

Slioussar’s (2018) findings are interesting, because they show that the plural feature of the attractor may not be enough for causing agreement attraction and that it is the identity of the form of the attractor with the nominative plural form (i.e., its case syncretism) what drives the attraction effects.

These findings on Russian led Lacina and Chromý (2022) to test similar structures on Czech in two experiments which were run in the same line of research as the one presented here. Crucially, the authors failed to find any effects of agreement attraction effects even in syncretic conditions. A similar finding was documented by Chromý et al. (2023) in a direct experimental comparison of Czech and English which was done in parallel to the present study. The authors showed that while an agreement attraction effect was clearly visible for English data, no such effect was attested for Czech translation equivalent stimuli. Moreover, an untimed acceptability judgment study on Czech (Lacina, 2023) also did not reveal any agreement attraction effects. It did however show that Czech comprehenders were sensitive to the agreement violations present in attraction sentences.

### Theoretical Explanations

Agreement attraction effects both in production and comprehension have been explained using several theoretical approaches. Among the most discussed in the literature are the Marking and Morphing model (Bock et al., 2001; Eberhard et al., 2005) and Cue-based Retrieval models (Engelmann et al., 2019; Lewis & Vasishth, 2005; Logačev & Vasishth, 2016; Parker et al., 2017; Vasishth et al., 2019; Yadav et al., 2021). In recent years, the Self-organized Sentence Processing account (Smith et al., 2018; Villata et al., 2018) has been also proposed to explain agreement attraction effects.

While all of these models provide differing explanations of the phenomenon of agreement attraction, they nevertheless agree that agreement attraction effects should not be limited to a specific language, but are expected across different languages. For a deeper discussion of various models of agreement attraction, we refer readers to the studies by Paape et al. (2021) and Yadav et al. (2023).

### Current Study

In the current study, we set out to test whether agreement attraction facilitatory interference effects could be found in the comprehension of Czech sentences. For these purposes, we conducted four experiments which targeted different types of constructions in which the effects of agreement attraction should be present given the theoretical predictions of cue-based models and previous research on other languages.

Experiments 1 and 2 targeted the retroactive type of interference (cf. Jäger et al., 2017). In the retroactive interference the attractor noun stands between the subject and the verb. We used Czech sentences very similar to sentences such as (8) or (9), which yielded clear number agreement attraction effects in English comprehension (Wagers et al., 2009).

(8)  The letter from the investigator(s) allegedly was/were received in San Francisco in late March.
(9)  The slogan on the poster(s) unsurprisingly was/were designed to get attention.

In contrast to the English sentences, we used the future-tense auxiliary, because the past-tense auxiliary in Czech is not only overtly marked for number, but also for grammatical gender. The choice of the future-tense auxiliary was thus driven by the aim to rule out the possibly intervening effects of grammatical gender. The two experiments differed from each other in the voice of the verb and in attractor animacy: in Experiment 1, the attractor noun was animate and the VP was active, whereas in Experiment 2, the attractor noun was inanimate and the VP was passive.

Experiment 3 aimed to examine the proactive type of interference. In this type of interference, the attractor appears prior to both the subject and the verb (such as (10)) analyzed by Avetisyan et al. (2020) and Wagers et al. (2009).

 (10)  The musician(s) who the reviewer praise(s) so highly will probably win a Grammy.

Finally, Experiment 4 focused on similar constructions as Experiment 1 (retroactive type of interference with animate attractors), but employed attractor nouns which were not only plural in the critical condition, but also homonymous (syncretic) with the nominative plural form. Thus, Experiment 4 tested the role of case syncretism on agreement attraction. Case syncretism in agreement attraction was previously studied on Russian in Slioussar (2018).

In all experiments, we tested configurations with singular subject heads. Ever since the early literature on attraction effects in both production and comprehension, it has been argued that sentences with singular subjects give rise to stronger effects (e.g., Eberhard, 1997). In this, we followed the groundbreaking study of Wagers et al. (2009) in order to maximize the probability of the attraction effect appearing.

All the experiments were run web-based using the word-by-word self-paced reading paradigm. This allowed us to gather much larger samples compared to what has typically been seen in studies on agreement attraction in comprehension.

### The Structure of Czech

To understand the experiments and their results in full, it is necessary to be aware of certain grammatical features of Czech which differentiate it not only from English, but also from other languages examined in the literature. Three features are of an utmost importance, namely its (a) highly complex inflectional system with overt case marking, (b) relatively free word order, (c) heavy reliance on formal agreement.

Czech is an inflectional language (Short, 1993). Czech nouns and adjectives (and most numerals and pronouns) have explicit marking of case, number, and grammatical gender. These grammatical functions are marked using morphological endings and a single ending is used for marking all three categories together. This distinguishes Czech for example from Armenian, studied by Avetisyan et al. (2020), which is morphologically agglutinative and uses two separate suffixes for marking number and case. The presence of overtly marked case presents a possible obstacle for agreement attraction effects—on the one hand, the subject tends to be overtly marked as such (i.e., has a nominative ending), on the other hand, the attractor tends to be overtly marked as a non-subject, that is, it has a certain non-nominative ending (which depends on the particular construction). Intuitively, this may help comprehenders to correctly retrieve the subject since it allows them to distinguish just by the form of the noun what is and what is not a plausible subject.

Czech has seven cases and two numbers. Thus, each noun has fourteen grammatical forms. However, Czech noun paradigms show a high level of syncretism, that is, the same endings are used in one paradigm for different cases and/or numbers. To illustrate this, let us look at two examples (taken from our experimental items):

(11)  Složk-a  pro archivářk-y
    file-nom.sg for archiver-acc.pl=nom.pl=gen.sg=voc.pl
    ‘A file for (female) archivers’
(12)  Dárek       od  návštěvník-a
    gift[nom.sg=acc.sg] from visitor-gen.sg=acc.sg
    ‘A gift from (male) visitor’

In the example (11), the first noun *složkα* ‘file’ has an unambiguously marked nominative case (the form is not syncretic). This is the sentence subject. The second noun *archivářky* ‘archivers’ has a form which is homonymous between accusative plural, nominative plural, genitive singular or vocative plural. This is a typical example of a syncretic form. The true grammatical function of the noun is disambiguated only by the context–in this case by the preposition *pro* ‘for’, which has to be followed by the accusative case.

The example (12) is rather different. Here, both nouns have a syncretic form. The form of first noun is syncretic between nominative singular and accusative singular. The form of the second noun is syncretic between genitive singular and accusative singular. The second noun is disambiguated based on the preposition *od* ‘from’, which requires the genitive case. However, the first noun cannot be disambiguated by the present context. Since Czech sentences are typically SVO (sentence-verb-object), the preferred analysis would be that this is a nominative (and therefore the subject). But one can come up with a sentence continuation in which the first noun would be an object in the accusative case, see (13):

(13)  Dárek   od  návštěvník-a  sežra-l-a  kočk-a
    gift[acc.sg] from visitor-gen.sg eat-pst-f.sg  cat-nom.sg
   ‘The cat ate the gift from (male) visitor’

Example (13) points to another crucial feature of Czech, namely to its relatively free word order. Different word orders may be used in Czech without a difference in sentence meaning. In Czech, all possible orders of subjects, verbs and objects are acceptable (i.e., SVO, SOV, OVS, OSV, VOS and VSO). However, the different word order types do not occur with the same frequency. Based on a corpus of 6101 transitive clauses in Czech, Siewierska and Uhlířová (1998) claim that the SVO word order is the most typical one (present in 63.1% of cases) followed by the OVS word order (14.6%). The frequency of each of the remaining word orders is lower than 10%. Thus, one may claim that generally, SVO is the preferred and most frequent word order. However, there might be other factors which influence word order choice, such as information structure and animacy. For example, we may assume that since subjects have a strong tendency to be animate (Dahl, 2008), SVO would be a more frequent word order in sentences beginning with an animate noun than in sentences beginning with an inanimate noun, such as example (13). However, to our knowledge, there has been no systematic research focused on such issues in Czech (but see similar argumentation in Jasinskaja & Šimík, in press).

The reason why word order may be an important factor influencing the presence of agreement attraction effects is as follows. Since Czech speakers can use different arrangements of the verb, the subject, and other NPs in the sentence and since case marking can be an unreliable guide in distinguishing between the syntactic function of the NPs when nominative-accusative syncretism is present, sentences may be often temporarily ambiguous at some point. An example might be a sentence starting with an inanimate noun which is often syncretic between the nominative and accusative in Czech. Readers might underspecify the representation of such sentences (i.e., they might leave it open whether the NP is in the nominative or accusative case). Assuming that, the agreement form that does not match the number specification of the preceding NP might not be seen as unacceptable, but simply as signaling that the syncrectic form of the NP should be resolved as the accusative case.

The last feature which we think to be important for the present purposes is the heavy reliance of Czech on formal agreement. As opposed to Czech, languages often employ semantic agreement (cf. Corbett, 2006). In (British) English, for example, nouns such as *the committee* which are formally singular, but refer to a plurality (i.e., are semantically plural) can be used either with a singular predicate (e.g., *has decided*) or with a plural predicate (e.g., *have decided*)—see Corbett (1979, 2006). The former possibility is an example of formal (syntactic) agreement, whereas the latter illustrates semantic agreement. It appears that the use of semantic agreement is influenced by the type of the agreeing constituent. Corbett (1979, 2006, 2009) presents an agreement hierarchy (or a hierarchy of agreement positions) which is a useful tool for estimating the use of semantic agreement as compared to syntactic agreement for a language. The hierarchy is as follows: attributive > predicate > relative pronoun > personal pronoun. Corbett (2009) claims that “as we move rightwards along the Agreement Hierarchy, the likelihood of agreements with greater semantic justification will increase monotonically” (p. 349). In Czech, the use of semantic agreement is rather restricted (Ziková & Caha, 2004). For example, Czech has many nouns which are formally plural, but semantically singular, such as *dveře* (‘door’), *plavky* (‘swimsuit’), or *záda* (‘back’), and also nouns which are formally singular, but semantically plural, such as *námořnictvo* (‘navy’), or *dobytek* (‘cattle’). Such nouns would be ideal candidates for semantic agreement. However, even for these nouns, it holds that the subject-verb agreement or relative pronoun agreement obligatorily matches their formal number specification, not their semantic number. It is simply not possible in Czech to refer to a door or a swimsuit with a singular pronoun or to refer to the navy or cattle using a plural pronoun.

The heavy reliance on formal (syntactic) agreement might be an important factor influencing the presence of agreement attraction effects. We will return to this while discussing the results of our experiments.

## EXPERIMENT 1

The aim of Experiment 1 was to replicate the findings on agreement attraction on English and other languages. Sentences with a similar structure to English *The letter from the investigator(s) allegedly was/were received in San Francisco in late March* (cf. Wagers et al., 2009) were used. Two variables were manipulated: (i) attractor number (singular or plural), and (ii) verb number (singular or plural). The subject was always in the nominative singular and importantly, the attractor noun was always animate and in the genitive case.

### Methods

#### Participants.

One-hundred thirty four undergraduate students of Charles University (113 women, 20 men and 1 preferred not to answer; mean age 23.1 years) participated in the experiment for course credits. One further participant was excluded based on the response accuracy criterion (see Data Analysis section below). All participants were native speakers of Czech.

#### Materials.

Twenty-four experimental items were used in Experiment 1. Each sentence contained eight words and began with a singular subject which was always inanimate except for one item (in which the animacy was overlooked by mistake), followed by the preposition *od* (‘from’), an animate attractor noun (singular or plural), a one-word particle (such as *bohužel* ‘unfortunately’), the future-tense auxiliary (singular or plural), a verb in infinitive form and two more words which concluded the sentence (for example an adjective and an object noun). An item example is shown in Table 1.

**Table 1.** Experimental item example from Experiment 1 together with glosses according to the Leipzig glossing rules (Comrie et al., 2008).

	1	2	3	4	5	6–8
a	Pohled	od	kamarád-a	určitě	bud-e	probouzet krásné vzpomínky
	postcard[nom.sg]	from	friend-gen.sg	surely	will-sg	arouse nice memories
b	Pohled	od	kamarád-ů	určitě	bud-e	probouzet krásné vzpomínky
	postcard[nom.sg]	from	friend-gen.pl	surely	will-sg	arouse nice memories
c	*Pohled	od	kamarád-a	určitě	bud-ou	probouzet krásné vzpomínky
	postcard[nom.sg]	from	friend-gen.sg	surely	will-pl	arouse nice memories
d	*Pohled	od	kamarád-ů	určitě	bud-ou	probouzet krásné vzpomínky
	postcard[nom.sg]	from	friend-gen.pl	surely	will-pl	arouse nice memories
‘A postcard from a friend(s) surely will-sg/pl arouse nice memories.’						

Besides the 24 experimental items, the experiment included 120 filler items. 24 of the fillers were stimuli from Experiment 2, and the remaining 96 fillers presented fully grammatical sentences in Czech with diverse length and syntactic structure. Another three sentences were used as practice items. Altogether, participants’ task was to read 147 sentences, out of which 24 were ungrammatical (half of the experimental items from Experiment 1 and half of the experimental items from Experiment 2; none of the practice or other filler items were ungrammatical).

Each sentence was followed by a yes–no comprehension question. For the experimental items, the questions had always the same structure and contained the identical verb and identical subject as in the presented sentence and differed in the targeted object. Half of the items had a comprehension question with “yes” as the correct answer, the other half with “no”. For example, for the item presented in the Table 1, the question was *Bude pohled probouzet vzpomínky?* ‘Will the postcard arouse memories?’. For the filler items, the questions varied and targeted various pieces of information from the sentences. Crucially, throughout the experiment, the ratio of questions with “yes” and with “no” as the correct answer was 1:1.

#### Procedure.

The experiment was web-based and conducted using the Ibex software on the IbexFarm platform (Drummond, 2011) using the moving window word-by-word self-paced reading paradigm.

When participants entered the experiment website by means of a provided link, they were first asked to read information about the study including a consent form, they received instructions for the experimental task and filled out a brief demographic questionnaire. Next, they read three practice sentences in order for them to become acquainted with the reading procedure. Following this practice session, the experiment commenced.

The participants were instructed to use the space bar to move from word to word in a moving display. Following the last word of each sentence, a comprehension question was displayed together with the binary option of “yes” or “no”. There was no time-out set for answering the comprehension question and no feedback as to the correctness of their answers was provided.

Each participant saw only one of the conditions of each item and the conditions were distributed based on the Latin-square design. Altogether, each participant was exposed to six exemplars of each condition. The order of sentences was fully randomized for each participant. The experiment took about 30 minutes.

#### Data Analysis.

Participants’ accuracy in answering comprehension questions was calculated and the participants whose accuracy scores to comprehension questions were 75% or lower were excluded from the subsequent analysis. Only one participant scored below 75% and was excluded based on this criterion. After the exclusion of this participant, the mean response accuracy was 97.2% [97%; 97.4%] for experimental items and 94.5% [94.4%; 94.6%] for fillers.

Before the analysis, reaction times (RT) outliers were removed. The minimum cut-off point was set to 100 ms, the maximum standard point was decided based on the visual inspection of the spread of RTs (following recommendations by Baayen & Milin, 2010). All RTs higher than 5,400 ms were removed. Altogether, 0.32% data points were removed. Remaining RTs were log-transformed for the subsequent analysis.

We examined the data in the Bayesian paradigm using hierarchical regression models (Gelman et al., 2013; Gelman & Hill, 2006; McElreath, 2016). We used the *brms* package (Bürkner, 2017) and the programming language R (R Core Team, 2022).

We ran three separate analyses: on the inflected verb (region 5, the word *bude*/*budou* ‘will’), the word following the verb (region 6, the word *probouzet* ‘arouse’) and the word after that (region 7, the word *krásné* ‘nice’). All the models on all three regions had the same structure.

In Bayesian data analysis, one specifies the likelihood, and the prior distributions over parameters of interest. The analysis results in posterior probability distributions of parameters for a given model and data. We report medians and 89% credible intervals (CrL), that is, the range of values for which we can be 89% certain that the true effect lays therein (see McElreath, 2016 for the discussion of 89% credible intervals). We also explore and present the Bayes Factor for the region in which the agreement attraction effect has commonly been observed in other studies (one word after the verb). See below for details.

Unless explicitly stated otherwise, models used 4 sampling chains with 4,000 samples drawn from each chain. Half of these samples were discarded for warm-up, hence each model had 8,000 samples available for the analysis. Only the models with all $\hat{R}$ ≤ 1.01, which suggests convergence, were used in the analyses. For the calculation of the Bayes Factor, 10,000 samples were drawn from each chain and 2,000 samples were discarded for warm-up.

#### The Structure of Bayesian Models.

The models took into account the factors of attractor number, verb number, and their interaction, which were coded using sum-contrast coding: the plural was coded as 1, the singular number was coded as −1. We assumed weakly informative priors for the model (Gelman et al., 2013; Schad et al., 2021). The dependent variable was RTs in log-ms. The following prior structure for the models was used:

- the intercept had a normal distribution with *μ* = 6 and *σ* = 1;
- the slopes for fixed effects and the standard deviation of the random effects had a normal distribution with *μ* = 0 and *σ* = 0.5 (truncated at zero for the latter);
- the residual standard deviation had a normal distribution with *μ* = 0 and *σ* = 1 truncated at zero;
- the random effects correlation had a LKJ distribution (Lewandowski et al., 2009; Stan Development Team, 2021) with *η* = 2.

The prior structure was virtually identical for the calculation of the Bayes Factor, but we varied the parameters of the prior that was the main goal of the investigation, namely the interaction attractor:verb (see below for details).

### Results

#### Descriptive Statistics.

**Figure 1.**
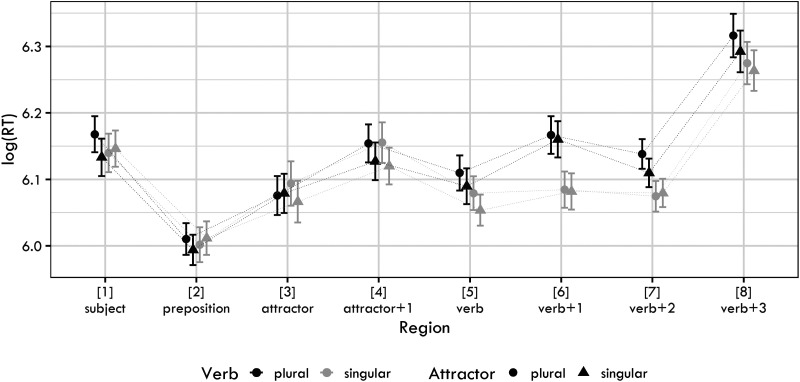
Mean log reaction times together with 95% confidence intervals for the four conditions used in Experiment 1.

Figure 1 shows the log-transformed RTs for each condition and sentence region. The raw RTs for the regions analyzed are in the Supplementary Materials.

#### Bayesian Modeling and Discussion.

**Figure 2.**
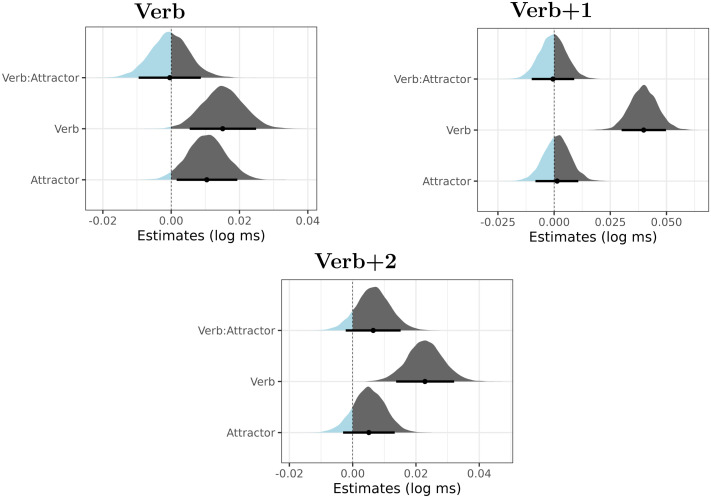
Posterior distributions of verb number (Verb), attractor number (Attractor) and their interaction in Experiment 1. The point is a median, the thick lines represent 89% credible intervals. The parts of the distribution that are below zero appear as light blue, the parts of the distribution that are greater than zero appear as dark gray.

The posterior distributions of the verb number, the attractor number factors and their interaction for the regions verb, verb + 1 and verb + 2 are summarized in Figure 2. The figures reveal a clear positive effect of verb number, showing that plural verb number led to a slowdown in the verbal and the post-verbal region. The slowdown is likely at least to some extent driven by the fact that sentences with plural verbs were ungrammatical, which would explain the robustness and persistence of the effect.

The attractor number also caused a slowdown (i.e., plural attractors caused slower reading times on the verb than singular attractors). In contrast to verb number, this effect was clearly visible only in Region 5 (i.e., the verbal auxiliary). This is likely a spillover effect of the plural attractor argument. Such a spillover effect has been documented in agreement attraction studies for other languages (e.g., Wagers et al., 2009).

Our main interest is the interaction term, which should be negative if agreement attraction was present. From visually inspecting the posterior distributions, it is clear that no such negative effect is present. However, to investigate the issue further, we calculate the Bayes Factor for two hypotheses: the “null” model (the model with the verb and the attractor as fixed terms, but without the verb:attractor interaction term) and the full model (the model with the verb, attractor and the verb:attractor interaction term). As is common, the null model only differs from the full model in the so-called fixed-effect structure, the random-effect structure is the same for both models.

We consider the Bayes Factor only for the region verb + 1. We selected this region as the most likely candidate to show the agreement attraction effect, since other self-paced reading studies observed the attraction effect on the first word after the verb (Avetisyan et al., 2020; Lago et al., 2015; Slioussar, 2018; Tucker et al., 2015; Wagers et al., 2009). We consider models with four different prior distributions for the interaction term. The first two models assume a normal distribution with the parameter values *μ* = 0, *σ* = 0.01, and *μ* = 0, *σ* = 0.03, respectively. Higher values than 0.03 for *σ* will unlikely lead to BF in favor of the model with the interaction term, given that even the very robust effect of verb number has the posterior distribution with median of 0.04. The third model and the fourth model are biased towards agreement attraction. That is, the prior for the interaction is shifted towards negative values. First, we consider the prior structure for the interaction term as the normal distribution with the parameter values *μ* = −0.03, *σ* = 0.009, which are rounded values used in previous studies on Bayes factor analysis of agreement attraction (Schad et al., 2022). It is also possible that Czech has agreement attraction but of a smaller magnitude than other languages. To test this, we include the last model which has the prior structure that is negative but less extreme: normal distribution with parameters *μ* = −0.015, *σ* = 0.0045.^2^

The Bayes Factors BF_01_ are summarized in Table 2. The values were collected using bridge sampling on models with four priors, described above.^3^

**Table 2.** Bayes Factors in Experiment 1 comparing the models without the interaction and with the interaction. Values higher than 1 indicate a change in evidence in favor of the model without the interaction, values lower than 1 indicate a change in evidence in favor of the model with the interaction.

*μ*	*σ*	mean BF_01_	5 sampled values of BF_01_
0	0.01	2.06	1.50, 2.04, 2.10, 2.17, 2.47
0	0.03	6.84	5.13, 5.93, 6.87, 7.13, 9.15
−0.03	0.009	75.1	49.77, 61.60, 63.64, 89.81, 110.51
−0.015	0.0045	7.13	5.16, 6.38, 7.21, 7.75, 9.14

BF_01_ values higher than 1 should be interpreted as showing a change in evidence in favor of the null model (i.e., the model without the interaction term), while BF_01_ values between 0 and 1 should be interpreted as showing a change in evidence in favor of the alternative model. In our case, the alternative model is the model with the interaction term. The more the value deviates from 1, the more support is gained for the null model or the alternative model. Following van Doorn et al. (2021), we say that values in the range [1, 3] and its inverse, [1/3, 1], are considered to be weak, ‘anecdotal’ evidence in favor of the null model and the alternative model, respectively. Values between [3, 10] and the inverse, [1/10, 1/3] are considered moderate evidence in favor of the null and the alternative model, respectively. Values higher than 10 represent strong evidence in favor of the null model and values smaller than 1/10 represent strong evidence in favor of the alternative model. As can be seen, every comparison is in favor of the null model, that is, the interaction-less model. The support for this model is weak when the prior for the interaction is centered around zero and assumes a narrow *σ* value. This is unsurprising since in this case, the model with the interaction term is hard to distinguish from the model that lacks the interaction term altogether. The evidence in favor of the null model is moderate and strong for the other priors. The evidence is the strongest with the interaction term parameters *μ* = −0.03, *σ* = 0.009.

The Bayes factor analysis shows that we have evidence of varying strength for the absence of the interaction in the model. Yet we have to be careful with the interpretation of this finding for linguistic models. It is possible that we did not observe any evidence in favor of the model with the interaction for other reasons than the absence of agreement attraction. More concretely, the Bayes factor analysis would reveal the preference for the interaction-less model not only if there was no attraction in ungrammatical sentences, but also if the plural attractor number caused a speed-up in both grammatical and ungrammatical sentences. Yet, it is the facilitatory interference of the plural attractor on the plural (ungrammatical) verb that is the hallmark of agreement attraction. This interference might go undetected should we only study the Bayes Factor comparing models with and without the interaction terms.

For this reason, we also calculated Bayes Factors for a subset of the data, namely, the ungrammatical conditions (in which the verb appears as plural), comparing the intercept-only model (the null model) with the alternative model, which has the intercept and one fixed factor, the attractor number condition. The random effect structure was identical for both models, consisting of intercept and attractor number condition for subjects and items. The same models were used in the Bayes Factor analysis of agreement attraction in Schad et al. (2022). In model comparison, we again consider four different normal distribution priors for the fixed factor of attractor number: (i) with parameters *μ* = 0, *σ* = 0.01, (ii) with parameters *μ* = 0, *σ* = 0.03, (iii) with parameters *μ* = −0.03, *σ* = 0.009, (iv) with parameters *μ* = −0.015, *σ* = 0.0045. The Bayes Factor values BF_01_ are summarized in Table 3.^4^

**Table 3.** Bayes Factors in Experiment 1 comparing the models without the attractor number factor and with the attractor number factor in ungrammatical sentences. Values higher than 1 indicate a change in evidence in favor of the model without the attractor number factor, values lower than 1 indicate a change in evidence in favor of the model with the attractor number factor.

*μ*	*σ*	mean BF_01_
0	0.01	1.53
0	0.03	3.81
−0.03	0.009	32.5
−0.015	0.0045	4.54

The comparison of models run on ungrammatical sentences paints the same picture as the comparison of models on the full data set. We again observe that all Bayes factors favor the null model and the weight of evidence in favor of the null model is the strongest for the prior with the parameters *μ* = −0.03, *σ* = 0.009.

In summary, we failed to find any signs of the agreement attraction effects. Importantly, we found ungrammaticality effects for the verb region, the verb + 1 region, and even the verb + 2 region. Such ungrammaticality effects have been found in other studies targeting agreement attraction for similar constructions (e.g., Patson & Husband, 2016; Wagers et al., 2009). The presence of such effects shows that participants processed the sentences carefully enough to notice the ungrammaticality.

There are two possible reasons for the absence of the attraction effects in this experiment. First, the attractor was overtly marked as genitive which would give the readers a cue not to consider that noun as a subject during retrieval. This would fit the view that in order to get agreement attraction effects, case syncretism of the attractor noun with the nominative plural form is needed (Slioussar, 2018). Second, the results may be related to the high reliance of Czech on formal agreement. Czech native speakers are simply not used to formal disagreement between subjects and verbs (this is also manifested by the long-lasting ungrammaticality effects we documented). Such an explanation would assume that Czech speakers either are better trained to retrieve the subject from memory, or they have a tendency to strongly adhere to the sentence subject which then gives them advantage in suppressing the interference from possible language material standing between the subject and the verb. Obviously, these two explanations are not mutually exclusive.

## EXPERIMENT 2

Experiment 2 was similar to Experiment 1 in that it examined the retroactive type of interference (cf. Jäger et al., 2017) with the attractor standing between the subject and the verb. However, there were two differences between the experiments: Experiment 2 employed solely inanimate attractors and the verbs were used in the passive voice. The reason for using inanimates and passives was motivated by the fact that the two experiments were conducted in one session and we wanted to avoid too many structurally identical items. In other words, our main aim was to examine another type of construction in Czech which might yield agreement attraction effects based on the findings from other languages.

### Methods

#### Participants.

Since Experiments 1 and 2 were conducted together in one session, the sample was identical to Experiment 1.

#### Materials.

In Experiment 2, we used 24 experimental items. This time, experimental stimuli contained ten words: (i) a singular subject (e.g., *regál* ‘a rack’), (ii) a preposition (e.g., *v* ‘in’), (iii) an inanimate attractor noun (e.g., *obchodě*/*obchodech* ‘shop/s’), (iv) a particle (e.g., *nepochybně* ‘surely’), (v) the future-tense auxiliary (*bude*/*budou*, ‘it will/they will’, (vi) an adverb (e.g., *pevně* ‘firmly’), (vii) a passive participle (e.g., *ukotven*/*y* ‘be anchored’), (viii) a sentence agent (e.g., *montérem* ‘by the assembler’), and two more words modifying the agent (e.g., *pochybné kvality* ‘of dubious quality’). Table 4 shows item example together with glosses.

**Table 4.** Experimental item example from Experiment 2 together with glosses according to the Leipzig glossing rules (Comrie et al., 2008).

	1	2	3	4	5	6	7	8–10
a	Regál	v	obchod-ě	nepochybně	bud-e	pevně	ukotv-en	montér-em pochybné kvality
	rack[nom.sg]	in	shop-loc.sg	surely	will-sg	firmly	anchor-pass[sg]	assembler-instr.sg dubious quality
b	Regál	v	obchod-ech	nepochybně	bud-e	pevně	ukotv-en	montér-em pochybné kvality
	rack[nom.sg]	in	shop-loc.pl	surely	will-sg	firmly	anchor-pass[sg]	assembler-instr.sg dubious quality
c	*Regál	v	obchod-ě	nepochybně	bud-ou	pevně	ukotv-en-y	montér-em pochybné kvality
	rack[nom.sg]	in	shop-loc.sg	surely	will-pl	firmly	anchor-pass-pl	assembler-instr.sg dubious quality
d	*Regál	v	obchod-ech	nepochybně	bud-ou	pevně	ukotv-en-y	montér-em pochybné kvality
	rack[nom.sg]	in	shop-loc.pl	surely	will-pl	firmly	anchor-pass-pl	assembler-instr.sg dubious quality
‘A rack in a shop(s) will-sg/pl surely be anchored-sg/pl by an assembler of dubious quality.’								

The experiment also included 120 filler times (24 of them were experimental stimuli from Experiment 1 and the rest of the fillers was identical to Experiment 1). Three sentences were used as practice items prior to the experiment. Participants thus read 147 sentences.

Each sentence was followed by a yes–no comprehension question. The comprehension questions used for the experimental items targeted the relation between the subject and the verb and never targeted the attractor. For example, for the item example from Table 4, a question *Ukotví někdo regál?* ‘Will someone anchor the rack?’ was used. Half of the questions for experimental items had “yes” as the correct response, the other half “no”. The structure of the questions for the filler items is explained in the Materials section of Experiment 1.

#### Procedure.

The procedure was identical to Experiment 1.

#### Data Analysis.

The same steps were followed as for the Experiment 1. The mean response accuracy for experimental items was 92.8% [92.5%; 93.1%].

### Results

#### Descriptive Statistics.

**Figure 3.**
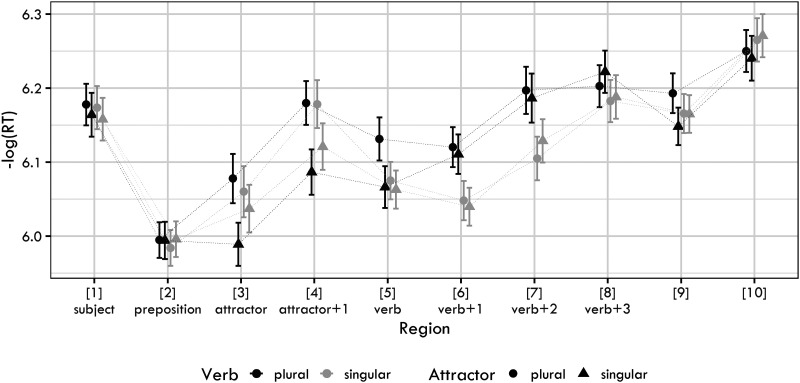
Mean log reaction times together with 95% confidence intervals for the four conditions used in Experiment 2.

Figure 3 shows the log-transformed RTs for each condition and sentence region. The raw RTs for the regions analyzed are in the Supplementary Materials.

#### Bayesian Modeling and Discussion.

**Figure 4.**
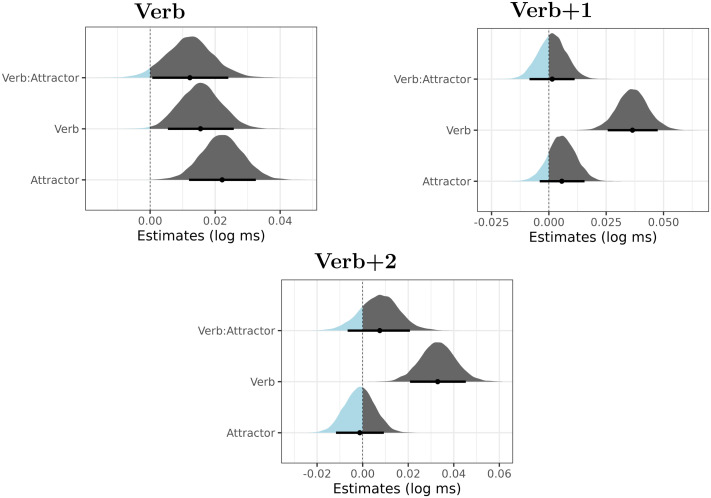
Posterior distributions of verb number (Verb), attractor number (Attractor) and their interaction in Experiment 2. The point is a median, the thick lines represent 89% credible intervals. The parts of the distribution that are below zero appear as light blue, the parts of the distribution that are greater than zero appear as dark gray.

Figure 4 summarizes posterior distributions for the verb, verb + 1 and verb + 2 regions.

The figure reveals that the major findings of Experiment 1 are replicated. We again documented long-lasting verb-number effects, which are most likely caused by ungrammaticality. We also see a slowdown due to plural attractors, most prominently present in the RTs of the verb.

A partial difference lies in the results for the verb region. In Experiment 1, the posterior distribution for the interaction term was not clearly skewed to the positive or the negative values. However, in Experiment 2, we see that the posterior of the interaction is predominantly positive. This interaction could be interpreted as a document of an *inhibitory* interference of the plural attractor. However, a more plausible explanation of this effect would be a combined influence of the spillover of the attractor number effect and the verb number ungrammaticality effect. In either case, it is worth pointing out that the interaction term goes in the opposite direction than we would expect if agreement attraction was at play.

As with Experiment 1, we proceed by the Bayes factor analysis for region 6 (i.e., the verb + 1 region). Two comparisons are made: (i) in the full data set, we compare the interaction-less model (the null model) and the full model, which includes the interaction term as a fixed effect (apart from this difference the two models are identical), (ii) in the ungrammatical subset, we compare the model with only the intercept (the null model) and the full model, which includes the intercept and the attractor number (apart from this difference the two models are identical).

We start with the first comparison. Bayes factors are summarized in Table 5. The results are similar to the results of Experiment 1. None of the prior distributions for the interaction term makes the full model preferred over the null model. The null model is favored more clearly when we assume a strong negative effect, with a normal distribution with the parameters *μ* = −0.03, *σ* = 0.009 as the prior for the interaction term.

**Table 5.** Bayes Factors in Experiment 2 comparing the models without the interaction and with the interaction. Values higher than 1 indicate a change in evidence in favor of the model without the interaction, values lower than 1 indicate a change in evidence in favor of the model with the interaction.

*μ*	*σ*	mean BF_01_	5 sampled values of BF_01_
0	0.01	1.77	1.24, 1.76, 1.93, 1.95, 1.99
0	0.03	6.79	5.30, 5.31, 6.94, 7.88, 8.51
−0.03	0.009	133.5	100.55, 101.95, 114.45, 152.10, 198.37
−0.015	0.0045	24.51	17.70, 23.1, 26.25, 27.24, 28.3

The Bayes factors for the second comparison (the ungrammatical subset) are summarized in Table 6. There is evidence in favor of the null model for all prior distributions. The strongest evidence in favor of the null model is present for the model with the normal prior distribution with the parameters *μ* = −0.03, *σ* = 0.009.

**Table 6.** Bayes Factors in Experiment 2 comparing the models without the attractor number factor and with the attractor number factor in ungrammatical sentences. Values higher than 1 indicate a change in evidence in favor of the model without the attractor number factor, values lower than 1 indicate a change in evidence in favor of the model with the attractor number factor.

*μ*	*σ*	mean BF_01_
0	0.01	1.4
0	0.03	3.14
−0.03	0.009	75.5
−0.015	0.0045	9.36

## EXPERIMENT 3

In the first two experiments, we found pronounced ungrammaticality effects for agreement discord structures and simultaneously, we failed to find any evidence for agreement attraction. In both experiments, we examined the retroactive type of interference in which the attractor stood between the subject and the verb.

In Experiment 3, we aimed to examine the proactive type of interference. In this type of interference, the attractor is presented before both the subject and the verb. Such structures have been examined in several languages such as English (e.g., Wagers et al., 2009), Spanish (Lago et al., 2015), or Armenian (Avetisyan et al., 2020). Two independent variables were manipulated: (i) matrix clause subject (i.e., attractor) number, (ii) relative clause verb number.

The experiment was preregistered on OSF here: https://osf.io/vnsp3.

### Methods

#### Participants.

Participants were drawn from the participant pool of the LABELS Lab which consisted of undergraduate students of Charles University. One participant was excluded for having lower than 75% response accuracy for filler items. The resulting sample consisted of 212 participants (183 female, 28 male, and 1 unspecified; mean age 22.57 years). All participants were native speakers of Czech and they participated for course credit.

#### Materials.

Twenty-eight experimental items were used. An example of an item is shown in Table 7. Each sentence comprised eleven words: (i) a matrix clause subject (singular or plural), (ii) a relative pronoun, (iii) a relative clause subject (always singular), (iv) an adverb, (v) a relative clause verb (singular or plural), (vi) a noun in the instrumental case, (vii) a matrix clause verb (always correctly agreeing with the matrix subject), (viii) a preposition *v* (‘in’) or *na* (‘on/at’), (ix) a noun denoting location (in the locative), and two more words which sensibly concluded the sentence. The embedded relative clause was separated by a comma from both sides (accordingly to the rules of Czech orthography).

**Table 7.** Experimental item example from Experiment 3 together with glosses according to the Leipzig glossing rules (Comrie et al., 2008).

	1	2	3	4	5	6	7	8–11
a	Závodník,	kter-ého	fanoušek	nezřídka	natáče-l	kamerou,	byl	v zemi velice oblíben-ý.
	racer[nom.sg]	who-acc.sg	fan[nom.sg]	often	record-pst[sg]	with camera	was[sg]	in country very popular-nom.sg
b	Závodníci,	kter-é	fanoušek	nezřídka	natáče-l	kamerou,	byl-i	v zemi velice oblíben-í.
	racer[nom.pl]	who-acc.pl	fan[nom.sg]	often	record-pst[sg]	with camera	was-pl	in country very popular-nom.pl
c	*Závodník,	kter-ého	fanoušek	nezřídka	natáče-l-i	kamerou,	byl	v zemi velice oblíben-ý.
	racer[nom.sg]	who-acc.sg	fan[nom.sg]	often	record-pst-pl	with camera	was[sg]	in country very popular-nom.sg
d	*Závodníci,	kter-é	fanoušek	nezřídka	natáče-l-i	kamerou,	byl-i	v zemi velice oblíben-í.
	racer[nom.pl]	who-acc.pl	fan[nom.sg]	often	record-pst-pl	with camera	was-pl	in country very popular-nom.pl
‘The racer(s) who the fan often recorded-sg/pl with a camera, was/were very popular in the country.’								

Apart from the experimental stimuli, the experiment included 98 sentences as filler items. Three sentences were used as practice items. In sum, participants read 129 sentences, out of which 14 were ungrammatical, i.e, half of the experimental items (none of the practice or other filler items were ungrammatical).

As in previous experiments, each sentence was followed by a yes–no comprehension question. For the experimental items, the questions always contained the embedded clause verb and either combined it with the subject of the embedded clause or with the subject of the matrix clause and the object was always unspecified using an indefinite pronoun. For example, the item in Table 7 was followed by the question *Natáčel někoho fanoušek?* ‘Did the fan record someone?’. Half of the items had “yes” as the correct response and other half “no”. The comprehension questions for filler items varied in their form and targeted various pieces of information.

#### Procedure.

The procedure was almost identical to the previous experiments. In contrast to the previous experiments, the experiment was hosted on the PC Ibex Farm (Zehr & Schwarz, 2018), because the previously used IbexFarm server (Drummond, 2011) had been shut down. Another difference between the experiments lied in the number of items per condition that each participant saw. This time, each participant received seven examples of each condition. The experiment was also slightly shorter. It typically took the participants about 25 minutes.

#### Data Analysis.

The same analytical steps were done as in the previous experiments. After the exclusion of one participant due to their low response accuracy (see Participants section), the mean response accuracy was 84.2% [83.9%; 84.5%] for experimental items and 94.8% [94.7%; 94.9%] for fillers.

Before the analysis, reaction times (RT) outliers were removed. The minimum cut-off point was set to 100 ms, the maximum standard point was decided based on the visual inspection of the spread of RTs. All RTs higher than 7,000 ms were removed. In sum, 1.16% data points were excluded from the analysis. Remaining RTs were log-transformed for the subsequent analysis.

The Bayesian model had the same structure and priors as models in Experiment 1 and Experiment 2.

### Results

#### Descriptive Statistics.

**Figure 5.**
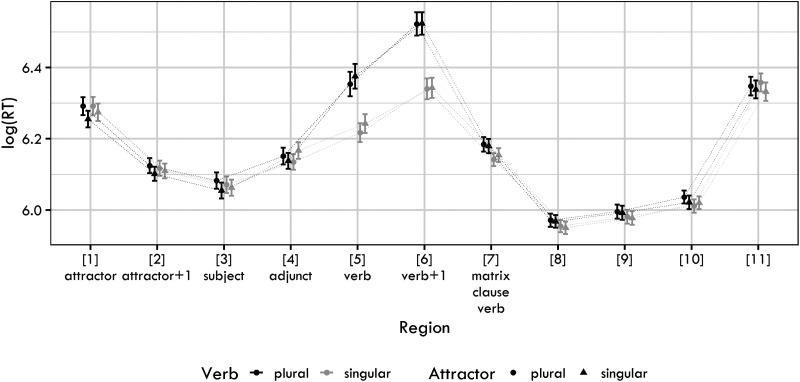
Mean log reaction times together with 95% confidence intervals for the four conditions used in Experiment 3.

Figure 5 presents the log-transformed RTs for each condition and sentence region. The raw RTs for the regions analyzed are in the Supplementary Materials.

#### Bayesian Modeling and Discussion.

**Figure 6.**
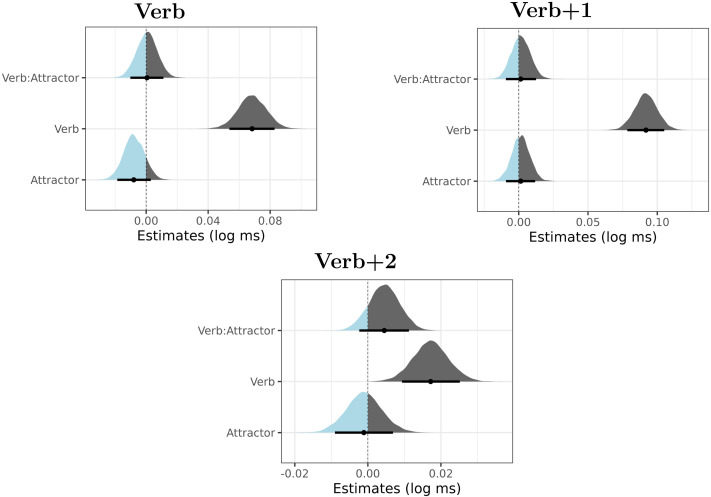
Posterior distributions of verb number (Verb), attractor number (Attractor) and their interaction in Experiment 3. The point is a median, the thick lines represent 89% credible intervals. The parts of the distribution that are below zero appear as light blue, the parts of the distribution that are greater than zero appear as dark gray.

The posterior distributions of the verb number, the attractor number factors and their interaction for the regions verb, verb + 1 and verb + 2 are summarized in Figure 6. The figures reveal a clear positive effect of the verb number. This replicates the ungrammaticality effect observed in the previous experiments.

In contrast to Experiment 1 and Experiment 2, the positive effect of the attractor number, however, is not visible in the figure. In the previous experiments, we suggested that the slowdown due to plural attractor number present on the verb might be a spillover effect since the attractor appeared just two words before the verb. In this experiment, the attractor appears at the beginning of the clause (Region 1) and thus, the lack of the positive effect is compatible with this explanation.

We next investigate the Bayes Factor. We start with the comparison of the models with and without the interaction term as a fixed effect.^5^ Four different models were considered, with varying prior distributions for the interaction term, just as in the previous experiments. Bayes Factors are summarized in Table 8.

**Table 8.** Bayes Factors in Experiment 3 comparing the models without the interaction and with the interaction. Values higher than 1 indicate a change in evidence in favor of the model without the interaction, values lower than 1 indicate a change in evidence in favor of the model with the interaction.

*μ*	*σ*	mean BF_01_	5 sampled values of BF_01_
0	0.01	0.26	0.07, 0.29, 0.30, 0.30, 0.33
0	0.03	1.7	0.71, 0.92, 1.36, 2.62, 2.90
−0.03	0.009	16.5	6.04, 14.54, 16.86, 21.09, 23.96
−0.015	0.0045	1.94	0.94, 1.37, 1.51, 2.1, 3.8

There is a preference for the null model (i.e., the model without the interaction term), in three out of four cases, even though the preference is less pronounced than in Experiments 1 and 2. In case of the model that assumes the prior normal distribution with the parameters *μ* = 0, *σ* = 0.01 for the interaction term, we see a (weak) change in evidence in favor of the full model (i.e., the model with the interaction term). Care has to be taken here. Even though there is a small preference for the full model in this case, this is not an argument for agreement attraction. The preference for the full model is likely driven by the fact that the interaction term is not null but slightly positive, which is the opposite sign than what we would expect if there was an agreement attraction effect. This explanation of the observed Bayes factor in the first row of Table 8 is in line with the fact that when we consider the full models that explicitly assume a negative interaction term, Bayes Factor is greater than 1, signaling a change in evidence in favor of the interaction-less model. This can be seen in the rows 3 and 4 in Table 8. Even an interaction term with the normal prior distribution very close to 0 (with parameters *μ* = −0.015, *σ* = 0.0045) does not show a change in evidence in favor of the model with the interaction term.

Next, we investigate the Bayes factor on the subset of the data, the ungrammatical condition, comparing the models with and without the attractor number manipulation as a fixed effect, just as in Experiment 1 and Experiment 2. The results are summarized in Table 9. The Bayes factors are slightly smaller than in Experiment 1 and Experiment 2 but they still consistently show a change in evidence in favor of the null model (the model without the attractor number as a fixed effect).

**Table 9.** Bayes Factors in Experiment 3 comparing the models without the attractor number factor and with the attractor number factor in ungrammatical sentences. Values higher than 1 indicate a change in evidence in favor of the model without the attractor number factor, values lower than 1 indicate a change in evidence in favor of the model with the attractor number factor.

*μ*	*σ*	mean BF_01_
0	0.01	1.47
0	0.03	3.44
−0.03	0.009	25.1
−0.015	0.0045	3.68

In conclusion, the proactive interference setup does not reveal agreement attraction effects. Such results differ from the findings of Avetisyan et al. (2020) on similar structures in Armenian. The authors of the study observed agreement attraction effects in their experiments and used them to argue that in languages with overt case marking, attraction effects are found even when case marking different from the subject-marking was present on the attractor. The present results (together with the results of Experiments 1 and 2), however, suggest that the phenomenon of agreement attraction may not be present in all languages that mark grammatical functions with overt case marking. We will return to this in the General Discussion.

## EXPERIMENT 4

In the first three experiments, we failed to find any signs of agreement attraction in Czech sentences, although the examined stimuli structurally resembled sentences previously found to yield attraction effects in other languages such as English, Spanish, or Armenian. In Experiment 4, we aimed to test the effects of case syncretism which has been argued to be a crucial feature of the attractor noun to getting agreement attraction effects in Russian (see Slioussar, 2018). We created sentences with retroactive interference (an attractor stood between the subject and the verb) and in which the attractor had the accusative case whose plural form was homonymous with the nominative plural.

The experiment was preregistered here: https://osf.io/kweaq.

### Methods

#### Participants.

Two-hundred sixty-three undergraduate students (222 women, 39 men and 2 did not disclose) of Charles University participated in Experiment 4 for course credit. Five additional participants were excluded because their response accuracy for filler items was lower than 75%. All participants were native speakers of Czech.

#### Materials.

We used 32 experimental items. Each sentence consisted of eight words: (i) a subject noun in the nominative singular, (ii) the preposition *pro* (‘for’), (iii) an attractor noun in the accusative case (singular or plural), (iv) an adverb, (v) the future-tense auxiliary *bude*/*budou* ‘will’ (singular or plural), (vi) a verb in the infinitive form, and two more words which sensibly concluded the sentence. An example of an item is shown in Table 10. Importantly, the plural forms of the attractor nouns were syncretic between nominative and accusative (i.e., the accusative plural form was homonymous with the nominative plural form). The singular form of the attractor was non-syncretic (it was uniquely accusative singular).

**Table 10.** Experimental item example from Experiment 4 together with glosses according to the Leipzig glossing rules (Comrie et al., 2008).

	1	2	3	4	5	6–8
a	Složk-a	pro	archivářk-u	nejspíš	bud-e	zahrnovat veškeré nálezy
	file-nom.sg	from	archiver-acc.sg	probably	will-sg	include all findings
b	Složk-a	pro	archivářk-y	nejspíš	bud-e	zahrnovat veškeré nálezy
	file-nom.sg	from	archiver-acc.pl=nom.pl	probably	will-sg	include all findings
c	*Složk-a	pro	archivářk-u	nejspíš	bud-ou	zahrnovat veškeré nálezy
	file-nom.sg	from	archiver-acc.sg	probably	will-pl	include all findings
d	*Složk-a	pro	archivářk-y	nejspíš	bud-ou	zahrnovat veškeré nálezy
	file-nom.sg	from	archiver-acc.pl=nom.pl	probably	will-pl	include all findings
‘A file for an archiver(s) will(-sg/pl) probably include all findings.’						

Another 96 sentences were used as filler items and three sentences were used as practice items in the beginning of the experiment. The filler items differed both in their length in words and their syntactic structures. In sum, participants read 131 sentences, out of which 16 were ungrammatical (i.e., the half of the experimental items). No other ungrammatical sentences were used in the experiment.

Each sentence was again followed by a yes–no comprehension question. The comprehension questions for experimental items targeted various pieces of information from the sentence, but never the attractor, because the attractor varied in number across conditions. For the filler items, comprehension questions were of various types and targeted various pieces of information.

#### Procedure.

The experimental procedure adopted was similar to previous experiments. PC Ibex Farm (Zehr & Schwarz, 2018) was used as in Experiment 3. Each participant received eight examples of each condition.

#### Data Analysis.

We follow the same analytical steps as in previous experiments. After the exclusion of five participants due to their low response accuracy (see Participants section), the mean response accuracy was 94.3% [94.1%; 94.4%] for experimental items and 94.2% [94.1%; 94.3%] for fillers. Clearly discontinuous RTs were trimmed (lower than 100 ms and higher than 5500 ms). In sum, 0.39% of data points were removed. For the Bayesian analysis, log-transformed RTs were used.

### Results

#### Descriptive Statistics.

**Figure 7.**
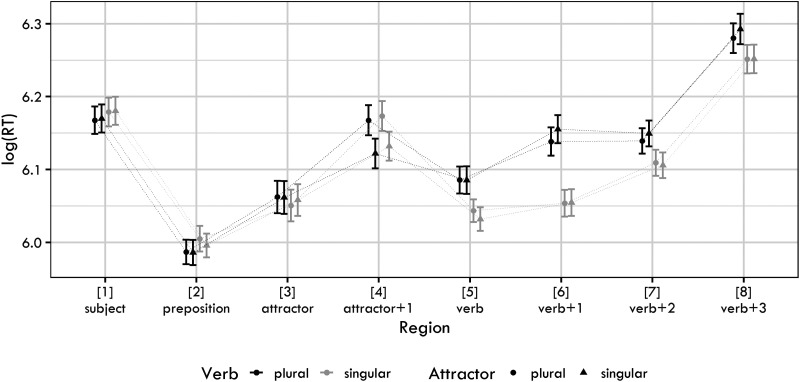
Mean log reaction times together with 95% confidence intervals for the four conditions used in Experiment 4.

Figure 7 graphically presents the log-transformed RTs for each condition and sentence region. The raw RTs for the regions analyzed are in the Supplementary Materials.

#### Bayesian Modeling and Discussion.

**Figure 8.**
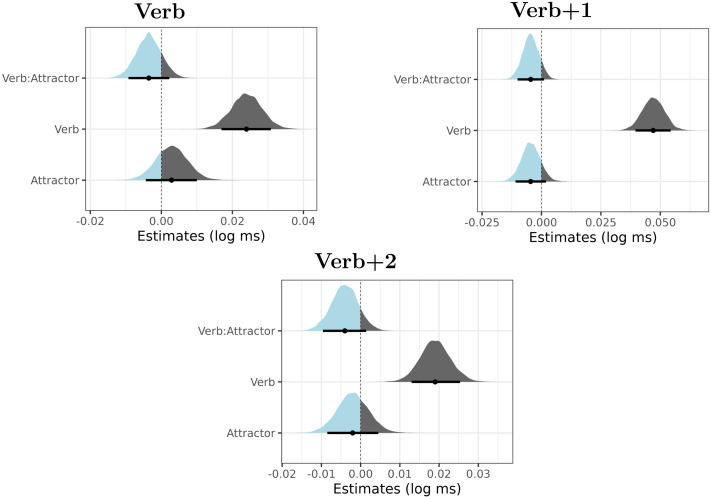
Posterior distributions of verb number (Verb), attractor number (Attractor) and their interaction in Experiment 4. The point is a median, the thick lines represent 89% credible intervals. The parts of the distribution that are below zero appear as light blue, the parts of the distribution that are greater than zero appear as dark gray.

The posterior distributions of the verb number, the attractor number factors and their interaction for the regions verb, verb + 1 and verb + 2 are summarized in Figure 8.

As in previous experiments, we see the effect of verb number, revealing the slowdown due to the ungrammatical plural verb.

There is also a visible negative interaction effect, even though the 89% credible interval includes zero. This is the first experiment in which such a negative shift in the interaction term is observed.

Bayes Factors comparing the null (interaction-less) model with four different full models, which all include the interaction term and vary its prior distribution, are given in Table 11. We can see that when the standard deviation is symmetrical around zero and very narrow, as in the row 1, there is no preference for either model (BF_01_ = 1.0). Otherwise the null model is preferred. This is even so when we consider the prior structure for the interaction term based on the meta-analysis of previous studies of agreement attraction, with *μ* = −0.03, *σ* = 0.009 (Jäger et al., 2017; Schad et al., 2022). The null model is preferred in this case since the interaction effect is too large. Even decreasing the mean of the interaction factor by half does not change the preference, Bayes factor still shows the preference, albeit very weak, for the null model.

**Table 11.** Bayes Factors in Experiment 4 comparing the models without the interaction and with the interaction. Values higher than 1 indicate a change in evidence in favor of the model without the interaction, values lower than 1 indicate a change in evidence in favor of the model with the interaction.

*μ*	*σ*	mean BF_01_	5 sampled values of BF_01_
0	0.01	1.0	1.3, 0.71, 0.83, 1.06, 1.11
0	0.03	9.2	5.62, 15.9, 11.54, 6.06, 6.75
−0.03	0.009	48.84	36.0, 44.48, 58.21, 57.89, 47.6
−0.015	0.0045	2.76	2.48, 4.33, 3.9, 2.9, 2.2

The Bayes Factor analysis shows that there is no change in evidence in favor of the model that includes the interaction, maybe with the exception of row 1, in which bridge sampling oscillates around the value of 1. This means that a clear evidence for facilitatory interference is absent.

We now turn to the Bayes Factor analysis on the ungrammatical subset, in which we compare the model with and without the attractor number as a fixed effect. The Bayes Factor values are summarized in Table 12. We see that the Bayes Factors support the alternative model in rows 1 and 4. These Bayes Factor values provide the first support for the alternative model even for the prior with a negative mean (see the BF_01_ value of 0.43 in row 4, which is identical to the BF_10_ value of 2.3). This is weak, ‘anecdotal’ evidence for the model with the attractor number as a fixed effect.

**Table 12.** Bayes Factors in Experiment 4 comparing the models without the attractor number factor and with the attractor number factor in ungrammatical sentences. Values higher than 1 indicate a change in evidence in favor of the model without the attractor number factor, values lower than 1 indicate a change in evidence in favor of the model with the attractor number factor.

*μ*	*σ*	mean BF_01_
0	0.01	0.58
0	0.03	1.25
−0.03	0.009	3.19
−0.015	0.0045	0.43

It is notable that the very small change in evidence in favor of the alternative model, seen in values smaller than 1 in row 1 and row 4 in Table 12 was only observed when a highly constrained prior was used. In particular, using a greater negative value for the prior (row 3) shows a change in evidence in favor of the null model. What this suggests is that the looked-for effect, if present at all, is very small, in fact, substantially smaller in Czech comprehension compared to what has been claimed to be the case in other languages. We will strengthen this last point in the next section, in which we study the size of the observed agreement attraction effect in Czech in the joint analysis of the four experiments and compare that to the size of the agreement attraction effect in other languages.

## JOINT ANALYSIS OF CZECH EXPERIMENTS 1–4 AND COMPARISON TO OTHER LANGUAGES

It is possible that even though the evidence for agreement attraction fails to materialize in individual experiments, aggregating the data from all four experiments would reveal more convincing evidence. For this reason, we merged the post-verbal reading data from the Experiments 1 to 4. The Bayesian models analyzing the data were similar to the ones considered in previous sections. Two versions of Bayesian models were considered: First, on the data set with all experimental conditions, we analyzed a model which included verb number, attractor number and their interaction as fixed effects as well as the full random structure for subjects and items of the four experiments. Second, we ran a model on the subset of the data that consisted of ungrammatical sentences only and we included the attractor number as the sole fixed effect and intercept and slope subjects and items random effects.

To repeat the main point of the paper, our claim is that Czech either shows no agreement attraction, or an agreement attraction effect is of a very small, almost negligible size. Thus our goal is not to just run the analysis of Experiments 1–4 but to compare the resulting Bayesian models to agreement attraction data from other languages.

For the close comparison with Czech, we had to select experiments and languages which (i) used the same methodology, that is, self-paced reading (otherwise, sizes of observed effects would be hard to compare), (ii) focused on the investigation of the same type of attraction, that is, the studies were solely focusing on number attraction, (iii) the design was comparable to Czech, that is, the experiments consisted of conditions in which verb number (singular/plural) was crossed with attractor number (singular/plural). If other conditions were considered in the experiment (e.g., subject number), we ignored the irrelevant conditions. That is, we ignored those conditions that our Experiments 1–4 did not test either.

We ended up with the following list of languages and experiments that served as the comparison to Czech number agreement attraction:

- Arabic: Tucker et al. (2015, 2021), Experiments 3, 4, and 5
- Armenian: Avetisyan et al. (2020), Experiments 2 and 3
- English: Wagers et al. (2009), Experiments 2, 4, and 5
- Spanish: Lago et al. (2015), Experiments 1 and 3

For each of these languages, we only analyzed reading data from the post-verb region, that is, the verb + 1 region, which was the locus of the Bayes factor analysis of the Czech data. If participants/items were removed in the original data analyses, we also removed them in our own analysis. However, for data trimming, we partly relied on our own procedure to mimic the analysis of the Czech data as closely as possible. This means that we removed reading times below 100 ms (original analyses had various cut-off points, varying from 100 to 200 ms). Since we used no single upper cut-off point across our own four experiments, we did not impose such an upper cut-off point on other languages, either. Rather, we relied on original analyses in this part.^6^

**Figure 9.**
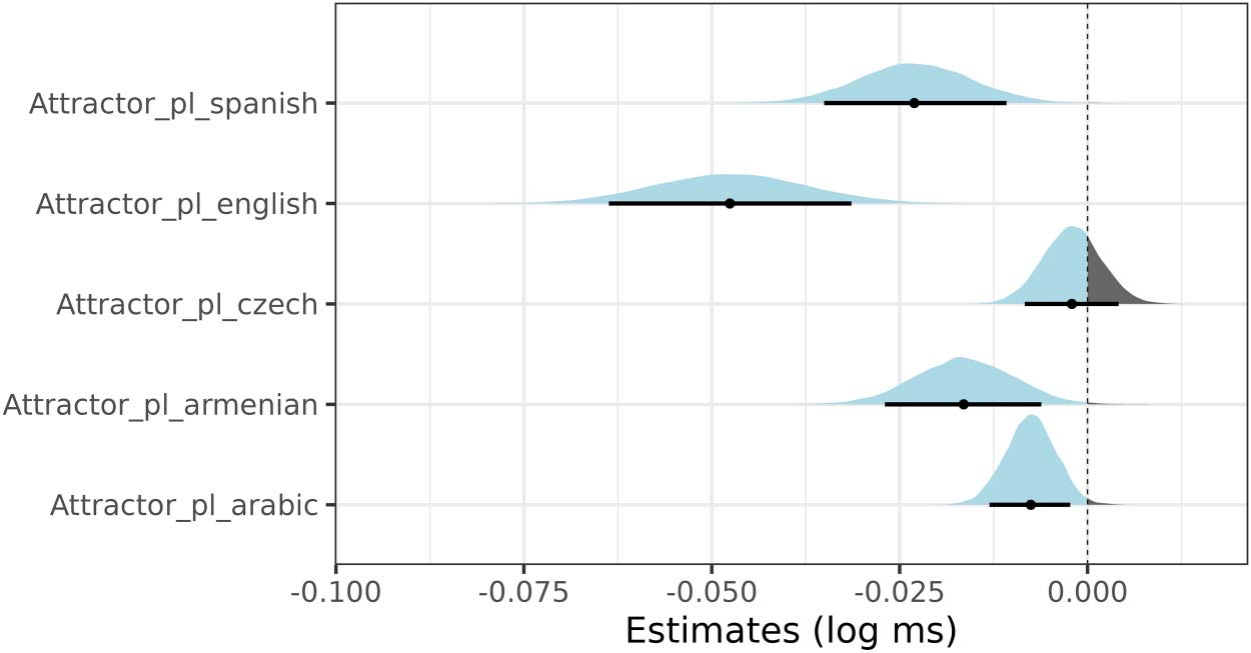
Posterior distributions and 89% credible intervals of plural attractor number effects in ungrammatical (verb plural) sentences in Czech, Arabic, Armenian, English, and Spanish. The data used for the analysis are discussed in the main text.

**Figure 10.**
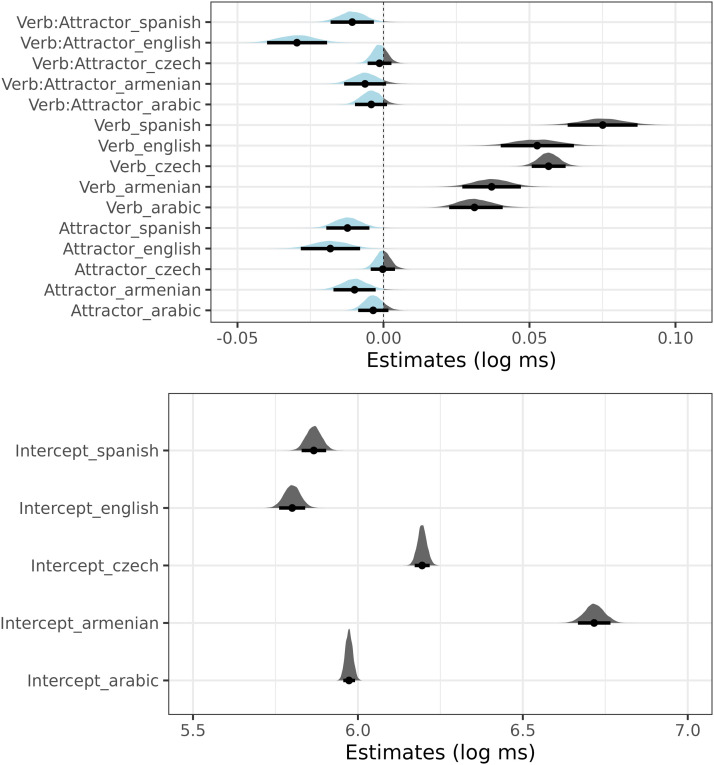
Posterior distributions of verb number (Verb), attractor number (Attractor) and their interaction (the top figure) and the intercepts (the bottom figure) in Czech, Arabic, Armenian, English, and Spanish. The data used for the analysis are discussed in the main text.

Posterior distributions of each language model are summarized in Figure 9 and Figure 10. The former figure summarizes the effect of attractor number (in log-ms) for the subset of the ungrammatical sentences, the latter figure summarizes posterior distributions (in log-ms) of the intercept, attractor number, verb number, their interaction in the full data sets.

Let us start with the first figure. Agreement attraction predicts a negative effect of the plural attractor number (since the analyzed ungrammatical sentences were only with plural verbs). While we can see that all five languages show negative values, languages clearly differ with respect to the size of the observed effect. In particular, Czech is the only language where the 89% credible interval clearly crosses the zero value. Crucially, this is not because the posterior distribution of the Czech agreement plural parameter would have a higher degree of uncertainty compared to other languages. In fact, the posterior distribution of the attractor plural number in Czech is, if anything, narrower compared to other languages, likely thanks to the fact that the experiments collected data from a large sample of 580 participants. The narrow distribution of the posterior distribution strongly suggests that the lack of observed agreement attraction in Czech is not likely due to more noise in our experiments caused by, for instance, their web-based nature. Rather, the comparison with other languages shows that agreement attraction, if present at all in Czech, is of a very small, almost negligible effect size.

The second figure, Figure 10, shows the posterior distributions of the intercept, attractor number, verb number and their interaction for the five analyzed languages. Just as in the previous figure, we see that the posterior distributions for the Czech data are narrower compared to other languages. We also see that the posterior distribution of the interaction term clearly crosses zero in Czech. In Spanish and English, the interaction term is evidently negative. In Armenian and Arabic, the posterior distributions are predominantly negative even though their 89% credible intervals include zero. Interestingly, the relatively small effect sizes of the interaction term in Armenian and Arabic correlate with the similarly small effect sizes of verb number (ungrammaticality) in the two languages compared to English and Spanish. This suggests that the small interaction terms might simply be the consequence of the fact that ungrammaticality led to a smaller increase in reading times compared to English and Spanish. Czech, in contrast, has a large effect size of verb number (ungrammaticality), yet the posterior distribution of its interaction term is almost zero and symmetrically spread around zero.

There is one possible confound that we should consider before concluding that the small to non-existent agreement attraction effect in Czech is supported by the merged analysis and the comparison with cross-linguistic data. The posterior distributions are presented in log-ms. Log is not a linear function. With the growth of the intercept, the same value of a parameter in log-ms will correspond to a greater difference in the non-transformed measure, ms. Thus, it is possible that the small effect size of the interaction term in Czech is simply the consequence of the fact that the intercept is high (i.e., reading times of the post-verbal region were slower in Czech than in the experiments conducted in the other languages). The bottom graph in Figure 10 shows that this is not the case. The intercept in Czech is much lower than the intercept in Armenian. Furthermore, the difference between the intercept in Czech and the intercept in English, Spanish and Arabic is not large enough to explain the much smaller effect size of the interaction term in the former.

In summary, the joint analysis of the Experiments 1–4 and the comparison with self-paced reading data from Arabic, Armenian, English and Spanish reinforce the conclusion that we arrived at for the individual experiments on Czech. We see that agreement attraction in Czech is absent or nearly absent. If it is present at all, it is of such a small magnitude that four experiments with 580 participants in total did not convincingly reveal the effect. The absence or near absence cannot be explained (away) as an artifact of this language not being sensitive to ungrammatical agreement, since the plurality of the verb in all four experiments lead to a slowdown comparable to English and Spanish. Finally, the narrow posterior distributions in the Czech data compared to the other languages strongly suggest that not finding an attraction effect should not be attributed to a larger uncertainty or variance in our data, but to the fact that the effect of number attraction on ungrammatical sentences in Czech is zero or nearly zero in the current experiments.

## GENERAL DISCUSSION

We studied number agreement attraction effects in Czech comprehension in four experiments.

The first three experiments revealed no relative speed-ups in reading times due to the matching attractor number in ungrammatical sentences. The effect was not visible in posterior distributions of Bayesian models. The Bayes factor analysis consistently and repeatedly showed evidence in favor of the null model, be it the model without the interaction when the full data set was considered, or the model without the fixed effect of attractor number when only ungrammatical sentences were considered. The evidence in favor of the null model was present for every considered prior structure in Experiments 1 and 2. In Experiment 3, the Bayes factor analysis showed evidence in favor of the model with the interaction when the prior for the interaction term was centered at zero and had a narrow distribution, *μ* = 0, *σ* = 0.01. This finding, however, was due to the inhibitory interference (i.e., the inverse of agreement attraction, as witnessed by the fact that the posterior distribution of the interaction term was positive and that the Bayes factor did not show the same preference when a negative prior for the interaction term was considered).

In Experiment 4, we saw a very small amount of evidence in favor of the model with the attractor number as a fixed effect for the ungrammatical sentences subset. Crucially, the evidence was present even when the prior distribution of the fixed effect was negative, with *μ* = −0.015, *σ* = 0.0045. Due to this finding, we cannot with confidence claim that our data give us reason to entirely rule out the presence of number agreement attraction effects in Czech comprehenders. The number agreement attraction effect might therefore be present in Czech. However, if it is present, its magnitude is extremely small at best.

To further investigate the last point, we pooled the data from all of our experiments and conducted a combined analysis. Additionally, we obtained and analyzed the raw data from several previous studies on agreement attraction from other languages, namely Arabic (Tucker et al., 2015, 2021), English (Wagers et al., 2009), Spanish (Lago et al., 2015), and Armenian (Avetisyan et al., 2020). This was done in order to compare the resulting posterior distributions of the size of the agreement attraction effect. What we saw was clear—agreement attraction was present in all of the languages with the exception of Czech. Next, the magnitude of the effect differed between the languages. English showed the largest effect size followed by Spanish. Armenian and especially Arabic, on the other hand, revealed effect sizes closer to zero. Czech was the only language whose 89% credible intervals of posterior distributions of the relevant parameter clearly crossed zero in the full data set as well as in the ungrammatical sentences subset. Crucially, the finding discussed in the previous sentence is not due to a large uncertainty in the posterior. Our Czech data set, as well as the Arabic data from Tucker et al. (2015, 2021), showed tighter credible intervals of the relevant parameter than other languages, likely due to the large pool of participants. The fact that our estimates are narrow grants us more confidence in claiming that Czech lacks anything but minuscule attraction effects. If the credible intervals in the joint analysis had turned out to be wide, it would be plausible to conclude that the failure to detect attraction was due to the excess of variability in the data. This, however, was not the case.

Let us now turn to the role played by case syncretism in agreement attraction effects. As shown by Slioussar (2018), whether the attractor noun has the same form as the nominative matters in both production and comprehension in Russian. Her results suggest that merely the sameness of the form of the attractor between the nominative plural and another case is sufficient for agreement attraction effects to appear. This was the case even when the attractor actually carried the singular feature. Likewise, Badecker and Kuminiak (2007) reported that in Slovak, a closely related language to Czech, case syncretism was a necessary condition for gender agreement attraction errors in production to arise.

In contrast to the results of Slioussar (2018), Lacina and Chromý (2022) report two self-paced reading experiments examining agreement attraction effects in comprehension in Czech. In them, they tested whether facilitatory interference in number agreement could be induced in the language by case syncretic attractors in the singular. They found that neither with feminine (Experiment 1) nor masculine (Experiment 2) subject heads was facilitatory interference observed. Similar to the current studies, only grammaticality slow-down effects were seen. The researchers tested both syncretic attractors in the singular and non-syncretic ones with the plural feature. In line with the current research, non-syncretic plural attractors also failed to show facilitatory interference in ungrammatical sentences.

In the current study, Experiments 1 to 3 did not employ syncretic attractors, but we tested the role of case syncretism was tested in Experiment 4. Unlike in the study of Lacina and Chromý (2022), we used case syncretic attractors that were also plural. It was in this experiment only that we saw some evidence in favor of facilitatory interference in number agreement. Should we take the presence of the effect as given, the question of how to explain the role of case syncretism arises. Our current data suggest that in Czech, case syncretism is jointly necessary together with plurality for the appearance of the effect, however tiny. This together with the results of Lacina and Chromý (2022) seems to differ from what we know about processing of agreement attraction in Russian. We speculate here that case syncretism might not have an independent effect on the presence of facilitatory interference (as Slioussar, 2018 argues is the case in Russian), but rather that both plurality and syncretism are jointly necessary to get at least some, although rather negligible effect.

Let us now turn to what the current results mean for the proposed theories attempting to explain agreement attraction facilitatory interference effects that we briefly discussed in the introduction. While all of the theories mentioned have differing predictions in specific configurations of targets, distractors and the features involved, they all agree on one thing—that in sentences in which the number feature of the subject is mismatched with that of the verb and there is an attractor that is of the number required by the verb, attraction effects should be observed. As argued above, our results amount to essentially no substantial agreement attraction effects in Czech. At the present moment, explanations of why this is the case, we believe, amount to speculation only, as more research is necessary to explore what exactly makes Czech comprehenders different from those of other languages where the effects have been documented. Nevertheless, we provide several thoughts on potential lines of inquiry with regards to how the models might accommodate our results.

Firstly, there is the Marking and Morphing model (Eberhard et al., 2005). Under this model, agreement attraction is said to be caused by faulty representations of the antecedents of the elements requiring agreement. The lack of effects in Czech here could mean that, for one reason or another, there is less tendency for disruptions in the process of number morphing compared to other languages. That the only case in which we see any evidence of attraction involved case syncretism (i.e., the ambiguity of one form corresponding to different structural features), could be an argument in favor of this explanation. However, due to the minuscule magnitude of the effect observed and the apparent difference from Slioussar’s (2018) results, it remains to be explained why even in case-syncretic situations, almost no substantial effects are observed.

Secondly, there is the Cue-Based Retrieval model (e.g., Engelmann et al., 2019; Lewis & Vasishth, 2005). According to this view, agreement attraction effects are caused by retrieval processes that make use of cues and feature match. Here, we speculate that our results could be accommodated by asserting that in Czech, the weights of the different types of cues are not the same as in the languages previously studied. In particular, the structural cue weight (i.e., c-command, subjecthood) could be higher. In the model of Engelmann et al. (2019), when the weight of this cue is increased, then, *ceteris paribus*, the magnitude of facilitatory interference in ungrammatical sentences with matching attractors decreases. Why structural cues are stronger in Czech would nevertheless still remain a question to be answered.

Finally, the Self-Organized Sentence Processing model (Smith et al., 2018) may explain the results somewhat similarly as the Cue-Based Retrieval model (meaning that the structural cue weight for Czech would be higher), but may argue that the interference between the subject and attractor is already lower during encoding, not only during the retrieval.

While the current study focuses on the issue of number agreement attraction in ungrammatical sentences that would be explained by cue-based models as facilitatory interference, we believe it is important to briefly address the other prediction of these models. Namely, they predict inhibitory interference in grammatical sentences (Jäger et al., 2017). As is the case with facilitatory interference in our study, our data do not provide support for inhibition to be present in the post-verbal region of the grammatical conditions of our stimuli. The status of inhibitory interference is more controversial than that of its facilitatory counterpart. For example, Wagers et al. (2009) only found facilitatory and no inhibitory interference in their study. Likewise, the metanalysis of Jäger et al. (2017) found evidence in favor of inhibitory interference in neither subject-verb agreement nor antecendent-reflexive structures. This is also in line with a recent meta-analysis and results of Laurinavichyute and von der Malsburg (2023), who found no evidence for inhibitory interference in grammatical sentences either in the hither-to published literature or in their own experiments using subject-verb agreement in English. Our study, therefore, sheds further doubt on these effects.

Let us now address some of the limitations of the current study. First, it must be noted that all of our experiments were web-based. However, for reaction times research (such as our own), it has been shown that there is no reliable difference between laboratory and web-based experiments (see Hilbig, 2016). Also, we used the same pool of participants we would otherwise use in a laboratory setting (i.e., the undergraduates of Charles University who participate for course credits), so the possible limitations connected to participant sources such as Amazon Mechanical Turk or Prolific do not apply here (see Sauter et al., 2020). Moreover, we used the response accuracy for filler items as a control measure of whether the participants were reading carefully. This shows us that only a very small number of participants did not focus enough on the experiments, a number comparably similar to what we would expect in the lab. Needless to say, the possible noise stemming from the web-based nature of the experiments should have been compensated by us testing many more participants than is typically done in agreement attraction research. The consequences of having a large sample of participants are clear from the comparison between languages in the pooled data analysis which suggests that the present results ought not to be attributed to a larger uncertainty or variance in our data due to the web-based nature of the experiments.

Second, the failure to find robust effects of agreement attraction in our experiments may be due to the population being tested. It may be so that the undergraduate students of Charles University are rather skilled and efficient readers who are not influenced in any way by interference of the type related to agreement attraction. We may only speculate whether the results would be different if we tested other populations of native Czech speakers. However, previous studies on agreement attraction effects in other languages also tested undergraduate students. Thus, we do not believe the difference in the findings of this study should be due to the reading capabilities of the tested sample.

Third, a possible source of confounds are the spillover effects of the number of the attractor. Indeed, plural attractors by themselves had a tendency to be processed more slowly compared to singular attractors in Experiments 1 and 2 (but not in Experiment 4) and there was an independent attractor number effect even in the verb region in these experiments. However, we believe the role of attractor number is only marginal if any in the crucial verb + 1 region where agreement attraction effects typically manifest themselves (e.g., Wagers et al., 2009). Importantly, this region is already the third region following the attractor and the attractor number effects themselves are relatively small in our experiments (especially compared to the ungrammaticality effects caused by plural verbs). Also, we do not see any clear signs of independent attractor number effects in any of the four experiments in the verb + 1 region. Thus, we believe the potential role of attractor number spillover effects did not influence our results with regards to any possible attraction effects, at least not substantially.

Fourth, our results relating to the ungrammaticality effects may be somehow confounded with the difference in length and/or frequency between the future tense auxiliaries used in the experiments. It is true that the singular (*bude*) form is more frequent than the plural (*budou*) form. However, both forms are extremely frequent in general—*bude* has a frequency of 1205 instances per million words in the SYN2020 corpus (Křen et al., 2020), *budou* has 405 instances per million. Using log-transformation which is known to predict the frequency effects better, there is almost no difference between the two. We thus do not believe it would be sensible to predict general processing differences based on frequency for these two forms which could explain robust and clear ungrammaticality effects found in all four experiments.

The difference in length between the two forms is also subtle. The plural form is one grapheme longer and in speech, the forms are almost the same depending on the phonological classification since /*ou*/ is a Czech diphthong and not two individual phonemes. We do not believe such a tiny length difference could have been the reason for getting robust ungrammaticality effects as we documented in all four experiments.

Our study raises several questions that ought to be addressed in future research. Firstly, the surprising lack of agreement attraction effects in comprehension opens up the question whether production studies would replicate this pattern. As we mentioned in the introduction, our corpus analysis showed only three examples which could be cases of number agreement attraction errors out of 7088 occurrences of the potentially relevant examples in the written corpus. A similar finding stems from the analysis of number agreement attraction using the spoken corpus of Czech ORAL v1 (Kopřivová et al., 2017). Interestingly, Badecker and Kuminiak (2007) studied the production of agreement errors in Slovak, a language mutually intelligible with Czech (Golubović & Gooskens, 2015), and were able to observe attraction effects. Two things are important, however: (1) Badecker and Kuminiak (2007) focused on gender attraction and not number attraction (a production study of number agreement errors is missing both for Czech and Slovak), (2) they used an attentionally demanding secondary task to make the elicitation task harder (due to the fact that they did not find any evidence of agreement errors in the pilot experiment without the secondary task; Kuminiak, personal communication). Thus, we assume the presence of number agreement attraction effects in (unconstrained) production in Czech would be of similar magnitude as we documented for comprehension—such effects would be extremely rare if any were observed at all.

## CONCLUSION

We attempted to replicate attraction effects in the processing of number agreement in the comprehension of Czech sentences. In sum, we found little evidence for the presence of this effect in Czech comprehenders. A further comparison with studies from other languages confirmed the picture that the agreement attraction in Czech number verb agreement, if present at all, is tiny at best. These finding have potential repercussions for the computational models of agreement attraction and highlight the importance of cross-linguistic investigations in psycholinguistics, even for phenomena that have been well-established in major languages such as English.

## ACKNOWLEDGMENTS

We would like to thank first and foremost all of our participants from Charles University. Next, our gratitude goes to the two anonymous reviewers of this article who helped improve it as well as two other reviewers who commented on a previous draft of this paper submitted elsewhere. We also received valuable comments from the ERCEL lab members as well as from the members of Shravan Vasishth’s lab at the University of Potsdam. Finally, we would like to thank the audiences of AMLaP2021, HSP2022 and PsychoSlav2022 where this research was presented.

## AUTHOR CONTRIBUTIONS

Jan Chromý: Conceptualization, Methodology, Resources, Data curation, Investigation, Writing – Original draft preparation, Writing – Review & editing, Visualization. Radim Lacina: Conceptualization, Methodology, Resources, Writing – Original draft preparation, Writing – Review & editing. Jakub Dotlačil: Methodology, Data curation, Formal analysis, Writing – Original draft preparation, Writing – Review & editing, Visualization.

## FUNDING INFORMATION

The first author was supported by Alexander von Humboldt Foundation and by the Charles University institutional program Cooperatio. The second author was supported by the German Research Foundation (DFG) as part of the Emmy Noether project awarded to Nicole Gotzner (Grant Nr. GO 3378/1-1). The third author was supported by the NWO grant VC.GW.17.112.

## DATA AVAILABILITY

Experimental items, data, and analysis code to all four experiments are available on OSF: https://osf.io/p47y6/.

## Notes

^1^ The precise query in CQL query language was [tag="N..S1.*"] [tag="R.*"] [tag="N..P.*"] [tag="V..P.*"] within <s/>.^2^ The *μ* = −0.03, *σ* = 0.009 values were based on the meta-analysis of previous research on agreement attraction in ungrammatical sentences (see Jäger et al., 2017 and Schad et al., 2022), which showed the mean effect size of attractor number in ungrammatical sentences to be −22 ms, with the 95% credible interval [−36 ms, −9 ms]. The values were transformed into the log-ms scale and rounded. The rounded mean of −0.03 log-ms corresponds to −24 ms (assuming the intercept of 6 log-ms and no other effects). The mean of −0.015 log-ms, also used in the last Bayes factor calculation, corresponds to −12 ms.^3^ While bridge sampling is flexible enough to compare models of various complexity (see Schad et al., 2022), its disadvantage lies in the fact that the Bayes Factor values can slightly vary even when a lot of samples are collected. For this reason, we ran bridge sampling multiple (5) times and report all sampled values, as well as the overall mean.^4^ Since the models have a simpler structure, bridge sampling is more stable. For this reason, we report just a single value for BF_01_ (the mean of five sampling runs).^5^ In Experiment 3, the sampling for Bayesian models used to calculate Bayes Factor had 4 chains and 15,000 iterations, of which 4,000 draws were discarded for warm-up.^6^ The original analyses of the Arabic data made use of winsorization rather than simple cut-off points to remove outliers. To not introduce another divergence, we cut-off reading times at 100 ms and 4,000 ms (the latter cut-off point was based on visual inspection). To be sure, we also analyzed winsorized data, but the results did not differ from the results presented below.
