# Supplementary material for: Number Agreement Attraction in Czech Comprehension: Negligible Facilitation Effects
Source: Open Mind (Camb). 2023 Oct 27;7:802–36. doi: 10.1162/opmi_a_00107 (PMC10631795; doi:10.1162/opmi_a_00107)
Supplement: Supplementary file 1 [file opmi-07-802-s001.pdf]

Number Agreement Attraction in Czech Comprehension: Negligible Facilitation Effects

Supplementary materials

Jan Chromá[a], Radim Lacina[b], and Jakub Dotlačil[c]

[1]Charles University

[2]Osnabrück University

[3]Utrecht University

ÂÍ

Table 1

Raw mean reaction times in ms (together with 95% confidence intervals) for the four conditions used in Experiment 1.

	attractor	verb	reg. 3	reg. 4	reg. 5	reg. 6	reg. 7	reg. 8
a	sg	sg	484.91 [464.7, 505.13]	496.24 [479.07, 513.42]	453.21 [439.8, 466.62]	476.56 [460.83, 492.29]	460.34 [448.11, 472.56]	589.47 [563.52, 615.42]
b	pl	sg	509.11 [483.67, 534.54]	528.92 [504.14, 553.7]	471.1 [455.99, 486.21]	480.56 [462.3, 498.83]	462.08 [448.75, 475.41]	604.89 [575.2, 634.59]
c	sg	pl	481.69 [464.53, 498.84]	505 [484.52, 525.47]	483.31 [463.36, 503.25]	516.29 [498.58, 534.01]	474.41 [462.37, 486.45]	612.42 [584.22, 640.63]
d	pl	pl	479.77 [462.22, 497.33]	517.36 [498.66, 536.05]	487.75 [472.22, 503.29]	521.48 [503.62, 539.35]	490.4 [477.3, 503.49]	631.28 [602.05, 660.51]

Table 2

Raw mean reaction times in ms (together with 95% confidence intervals) for the four conditions used in Experiment 2.

	attractor	verb	reg. 3	reg. 4	reg. 5	reg. 6	reg. 7	reg. 8
a	sg	sg	479.01 [453.42, 504.59]	511.26 [489.62, 532.9]	467.12 [448.61, 485.63]	452.27 [438.46, 466.07]	507.6 [488.33, 526.88]	537.6 [517.64, 557.56]
b	pl	sg	497.75 [471.84, 523.66]	546.89 [522.12, 571.65]	469.15 [453.1, 485.2]	460.1 [443.31, 476.89]	496.85 [477.15, 516.55]	531.88 [512.8, 550.97]
c	sg	pl	444.88 [424, 465.77]	494.58 [472.11, 517.04]	473.39 [456.26, 490.53]	488.73 [472.56, 504.9]	559.23 [530.34, 588.13]	551.48 [532.87, 570.09]
d	pl	pl	503.93 [477.46, 530.4]	533.82 [513.69, 553.94]	509.98 [489.37, 530.59]	495.04 [478.15, 511.92]	557.45 [531.94, 582.95]	543.89 [522.59, 565.19]

Table 3

Raw mean reaction times in ms (together with 95% confidence intervals) for four conditions used in Experiment 3.

	attractor	verb	reg. 5	reg. 6	reg. 7	reg. 8
a	sg	sg	605.47 [581.88, 629.06]	681.71 [654.33, 709.1]	513.41 [498.87, 527.96]	412.09 [400.91, 423.27]
b	pl	sg	589.3 [565.54, 613.06]	690.95 [660.42, 721.48]	499.8 [487.65, 511.94]	410.35 [401.95, 418.75]
c	sg	pl	775.57 [736.63, 814.5]	850.72 [813.58, 887.85]	530.98 [514.8, 547.17]	419.55 [409.09, 430.02]
d	pl	pl	760.44 [720.63, 800.24]	859.4 [820.99, 897.8]	534.72 [518.09, 551.34]	423.55 [412.6, 434.5]

Table 4

Raw mean reaction times in ms (together with 95% confidence intervals) for four conditions used in Experiment 4.

	attractor	verb	reg. 5	reg. 6	reg. 7	reg. 8
a	sg	sg	449 [440.1, 457.89]	470.68 [460.16, 481.21]	491.06 [480.77, 501.34]	586.98 [570.31, 603.66]
b	pl	sg	451.32 [443.34, 459.31]	472.09 [460.25, 484.93]	493.85 [482.96, 504.74]	587.26 [570.59, 603.93]
c	sg	pl	489.03 [477.61, 500.44]	524.61 [512.52, 536.7]	514.8 [503.34, 526.26]	622.09 [603.41, 640.78]
d	pl	pl	487.52 [475.72, 499.33]	516.85 [504.75, 528.95]	505.87 [495.41, 516.32]	607.65 [590.67, 624.63]
